# Loss of habenular *Prkar2a* reduces hedonic eating and increases exercise motivation

**DOI:** 10.1172/jci.insight.141670

**Published:** 2020-12-03

**Authors:** Edra London, Jason C. Wester, Michelle Bloyd, Shelby Bettencourt, Chris J. McBain, Constantine A. Stratakis

**Affiliations:** 1 Section on Endocrinology and Genetics and; 2 Section on Cellular and Synaptic Physiology, *Eunice Kennedy Shriver* National Institute for Child Health and Human Development, NIH, Bethesda, Maryland, USA.

**Keywords:** Metabolism, Neuroscience, Behavior, Obesity, Signal transduction

## Abstract

The habenula (Hb) is a bilateral, evolutionarily conserved epithalamic structure connecting forebrain and midbrain structures that has gained attention for its roles in depression, addiction, rewards processing, and motivation. Of its 2 major subdivisions, the medial Hb (MHb) and lateral Hb (LHb), MHb circuitry and function are poorly understood relative to those of the LHb. *Prkar2a* codes for cAMP-dependent protein kinase (PKA) regulatory subunit IIα (RIIα), a component of the PKA holoenzyme at the center of one of the major cell-signaling pathways conserved across systems and species. Type 2 regulatory subunits (RIIα, RIIβ) determine the subcellular localization of PKA, and unlike other PKA subunits, *Prkar2a* has minimal brain expression except in the MHb. We previously showed that RIIα-knockout (RIIα-KO) mice resist diet-induced obesity. In the present study, we report that RIIα-KO mice have decreased consumption of palatable, “rewarding” foods and increased motivation for voluntary exercise. *Prkar2a* deficiency led to decreased habenular PKA enzymatic activity and impaired dendritic localization of PKA catalytic subunits in MHb neurons. Reexpression of *Prkar2a* in the Hb rescued this phenotype, confirming differential roles for *Prkar2a* in regulating the drives for palatable foods and voluntary exercise. Our findings show that in the MHb decreased PKA signaling and dendritic PKA activity decrease motivation for palatable foods, while enhancing the motivation for exercise, a desirable combination of behaviors.

## Abstract 2

Decreased habenular PKA signaling and altered localization of PKA catalytic subunits in medial habenula dendrites caused by Prkar2a deletion led to increased voluntary running and decreased sucrose solution intake in mice.

## Introduction

In the face of the global obesity epidemic, it remains unclear what makes some individuals more susceptible to obesity than others. Years of cumulative data show that the seemingly simple idea of balancing caloric intake with energy expenditure is complex and influenced by many opposing drives that are exacerbated by overscheduled sedentary lifestyles, changes in the food supply, and genetics (1). As a major player in the regulation of the midbrain monoaminergic system (2), the habenula (Hb) is a central structure that integrates rewards with cognition and emotion (3). While these Hb functions have been investigated in the context of substance abuse, a role for the Hb in obesity and susceptibility to the energy imbalance that drives preventable metabolic dysregulation is less clear.

To date, medial Hb (MHb) research has primarily centered on addiction (4–6) and mood-related disorders (7, 8). The MHb integrates the dysregulated rewards signaling that underlies the imbalance between reward-seeking and avoidance behaviors in depression and substance abuse (8, 9), and although this circuitry may be important to obesity, it has not been systematically investigated. While lateral Hb (LHb) connectivity and function have been more extensively studied (10, 11), it is increasingly evident that there may be complementary or synergistic roles for the MHb and LHb in regulating stress response, nociception, rewards, locomotor activity, and food intake. In the regulation of hedonic eating, the LHb mediates inhibition of palatable food intake through glutamatergic neurons that project from the lateral hypothalamus (12). Crosstalk between the MHb and LHb is unidirectional from the MHb to LHb (13), and while the interpeduncular nucleus (IPN) is a primary output target of both the LHb and MHb, each innervates distinct IPN structures (14, 15).

The MHb is subdivided into dorsal (dMHb) and ventral MHb subnuclei (vMHb), which can be identified by high expression of substance P or acetylcholine, respectively (16–18). Developmental elimination of dMHb neurons via deletion of the transcription factor *Pou4f1* blunted sucrose preference (19), but a deeper understanding of which cell populations in the MHb can regulate food rewards or signal satiety is lacking. We show here that *Prkar2a*, which codes for the cAMP-dependent protein kinase (PKA) regulatory subunit IIα (RIIα), is highly expressed in the MHb in a region that overlaps the dMHb and vMHb subnuclei and is therefore present in both acetylcholine- and substance P–expressing cells.

The few animal studies investigating PKA signaling in the MHb have demonstrated neuroendocrine functions via both pre- and postsynaptic modulation of PKA activity (20–22). In MHb axons, selective inhibition of PKA reversed the induction of glutamate release by atrial natriuretic peptide that plays a role in stress-induced analgesia (22). Additionally, the modulation of local cAMP levels in MHb neuronal nicotinic acetylcholine receptors (nAChRs) regulates nicotine intake through the diabetes-associated gene transcription factor 7 like 2 (5). These studies provide clear and divergent evidence for regulatory roles for PKA in the Hb, but the breadth of knowledge about specific roles of PKA activity or its inhibition in the MHb is incomplete. In mice, deletion of *Prkar2a* led to a diet-induced obesity–resistant (DIO-resistant) phenotype and improved glucose tolerance after chronic high-fat diet (HFD) feeding (23). There was no detectable metabolic phenotype under normal feeding conditions (23, 24). The observed DIO resistance, which was more prominent in female mice, could not be fully explained by altered metabolic rate that was only modestly increased after HFD exposure (23) but instead appeared to be the result of decreased HFD intake. Here, we explore how *Prkar2a* might regulate behaviors related to food intake, the motivation for natural rewards, and energy expenditure and how PKA signaling in the MHb might be altered by the deletion of *Prkar2a*.

## Results

### Prkar2a expression is localized to both substance P– and acetylcholine-expressing cells in the MHb.

**Figure 1:**
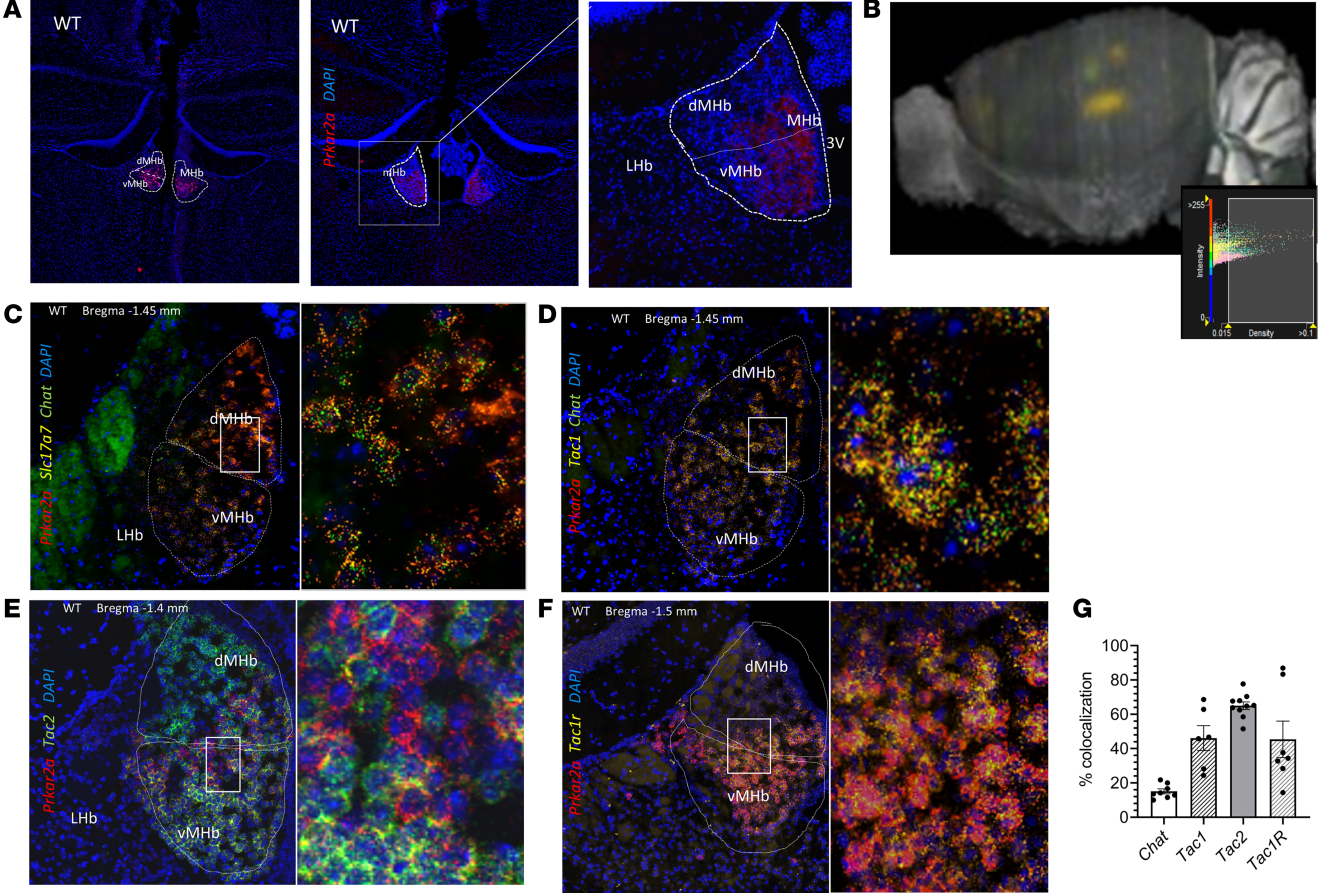
In brain, *Prkar2a* expression is nearly exclusive to Hb. (**A**) In situ hybridization (ISH) for *Prkar2a* (left) and immunofluorescence (IF) for PKA RIIα protein (right, original magnification 20×) with higher magnification (60×) detail show that expression in WT (C57BL/6) mouse brain is primarily in the MHb, and (**B**) 3D ISH for *Prkar2a* shows robust and specific habenular localization of *Prkar2a* (3D image: Allen Brain Institute). Representative ISH images (20×, and the higher magnification details, 60×) show colocalization of *Prkar2a* with (**C**) *Slc17a7* and *Chat*, (**D**) *Tac1* and *Chat* and (**E**) *Tac2*, and (**F**) *Tac1r* in dMHb and vMHb. (**G**) In MHb, *Chat*, *Tac1*, *Tac2*, and *Tacr1* were expressed in approximately 15%, 46%, 65%, and 40% of *Prkar2a*-expressing cells; *n* = 6–11 sections from 3 different mice (1 male, 2 female) for each target; 2-tailed unpaired *t* tests; *P < 0.05. All data represent the mean ± SEM.

Combinations of 2 PKA catalytic (Cα, Cβ, Cγ) and 2 PKA regulatory (RIα, RIβ, RIIα, RIIβ) subunits form the PKA holoenzyme; isoform composition of the tetrameric enzyme is tissue specific and affects cAMP binding affinity and cellular localization. All subunits except for RIIα are highly expressed in brain, yet expression for each appears to be specific (25) and largely nonredundant. At both RNA and protein levels, RIIα was expressed in both the dMHb and vMHb and around the junction of the 2 subnuclei (Figure 1A). The virtual absence of *Prkar2a* in other brain regions is evident from whole-brain-view, 3D ISH data (Figure 1B, Allen Brain Institutes). Because the habenular structure changes morphologically anterior to posterior, we mapped the expression of *Prkar2a* throughout the Hb from bregma –1.0 mm to –2.0 mm and found that *Prkar2a* expression peaked between bregma –1.3 to –1.7 mm (Supplemental Figure 1A; supplemental material available online with this article; https://doi.org/10.1172/jci.insight.141670DS1). These mapping studies also showed that *Prkar2a* expression patterns throughout the dMHb and vMHb varied from anterior to posterior points in the MHb. We used ISH with probes for *Syn-1* and *Gfap* to establish *Prkar2a* expression in both neuronal and glial cells of the MHb (Supplemental Figure 1B). Both MHb and LHb neurons use glutamate as their primary neurotransmitter (26, 27).

We initially confirmed that *Prkar2a* expression was limited to glutamatergic neurons and not expressed in a subset of GABAergic cells in WT mice by ISH using probes for *Slc17a7* and *Gad1* (Supplemental Figure 1C). In line with our expression studies, *Prkar2a* was the only PKA regulatory subunit identified as a highly and differentially expressed gene among the identified cell subsets within the MHb via single-cell transcriptome analysis of mouse (28). High relative expression of *Slc17a6* and *Slc17a7*, which code for vesicular glutamate transporters 1 and 2, and *Tac2* (coding for tachykinin precursor 2) was a common feature among 5 distinct neuronal cell populations identified in the MHb (28). To characterize the subsets of *Prkar2a*-expressing glutamatergic cells, we performed ISH with probes for choline acetyltransferase (*Chat*), *Tac1*, *Tac2*, and Tac1 receptor (*Tacr1*). *Prkar2a* was colocalized with *Chat* primarily in the vMHb (Figure 1, C and D) and with *Tac1* in the dMHb and to a lesser extent in the vMHb (Figure 1D). *Tac2* was distributed throughout the dMHb and vMHb and was expressed in 65.02% ± 2.20% of *Prkar2a*-expressing cells (Figure 1, E and G). Similarly, *Tac1* was expressed in 46.11% ± 7.26% *Prkar2a*-expressing cells in the MHb, where these genes are highly and specifically expressed (Figure 1, D, E, and G). *Tacr1* was expressed in 39.99% ± 7.01% in *Prkar2a*-expressing cells (for which substance P is the substrate) (Figure 1, F and G), and *Chat* was expressed in 15.10% ± 1.45% of the *Prkar2a*-expressing cells (Figure 1, C, D, and G). The colocalization studies showed that *Prkar2a* is expressed in a heterogenous population of MHb cells and is expressed in both substance P– and acetylcholine-producing neurons.

### Habenular PKA enzymatic activity is decreased in RIIα-KO mice.

**Figure 2:**
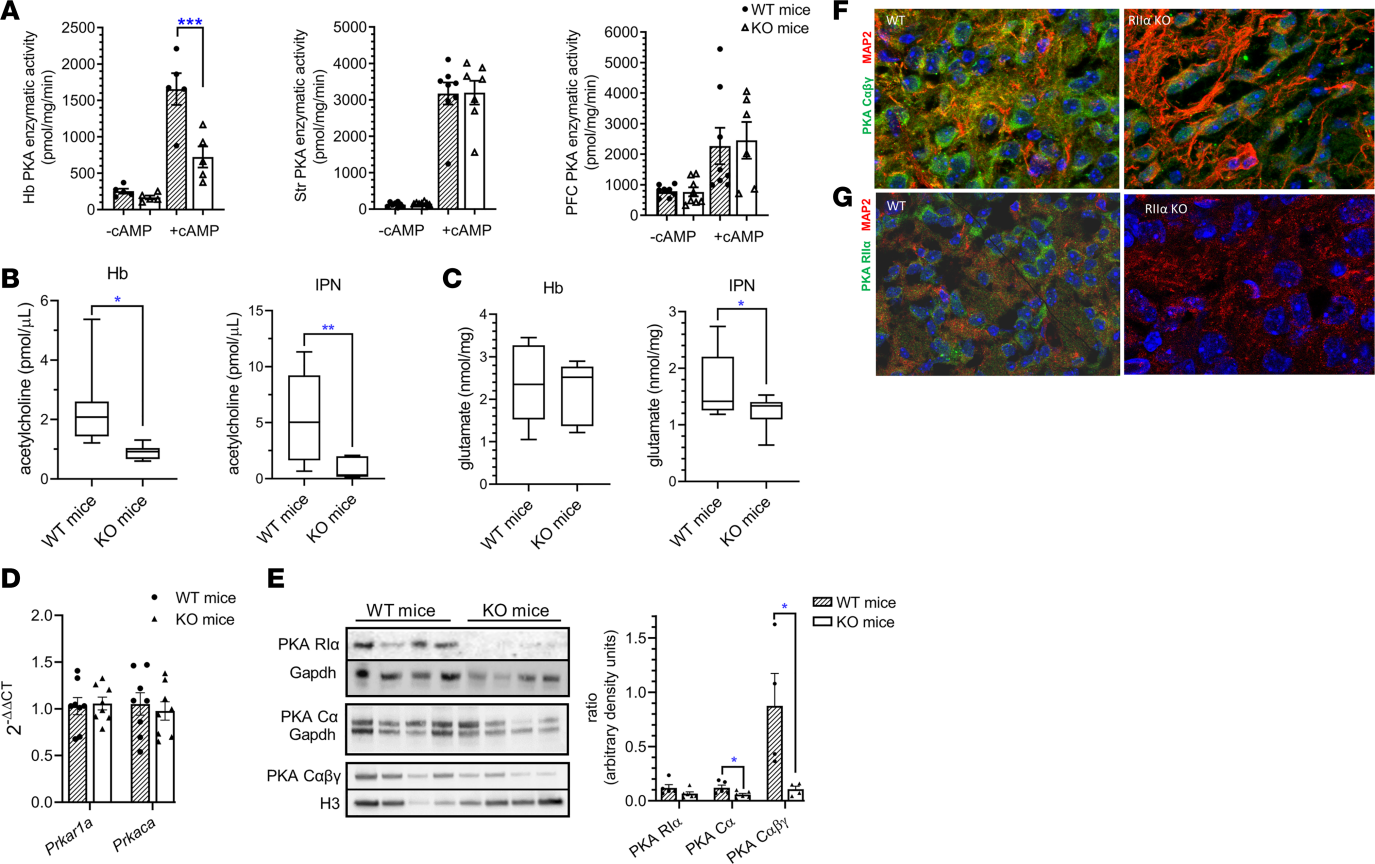
RIIα-KO mice had reduced habenular PKA enzymatic activity, decreased IPN acetylcholine and glutamate levels, and altered localization of PKA catalytic subunit to dendrites compared with WT mice. (**A**) Basal and total (cAMP-stimulated) PKA enzymatic activities in Hb, striatum (Str), and prefrontal cortex (PFC); *n* = 5–8/group (female data shown). (**B**) Acetylcholine concentrations in Hb and IPN were lower in RIIα-KO mice compared with WT littermates; *n* = 6–7/sex/group (male data shown). (**C**) Glutamate concentrations did not differ in Hb but were lower in IPN of KO compared with WT mice; *n* = 7–9/group (female Hb and male IPN data shown). (**D**) Habenular *Prkar1a* and *Prkaca* mRNA levels did not differ between WT and RIIα-KO mice; *n* = 7/group (male data shown). (**E**) Representative Western blots of Hb lysates for PKA subunits RIα and Cα (first membrane with Gapdh as housekeeper, females) and combined Cαβγ (second membrane with Histone 3 as housekeeper, males); *n* = 4/genotype. Representative immunofluorescent images of WT (left) and RIIα-KO (right) brain sections (MHb) showed differences in the subcellular localization of (**F**) PKA catalytic subunits (αβγ, green) in lower dMHb in WT, and mutant mice that had impaired dendritic localization (shown by staining for MAP2, red), and (**G**) PKA RIIα (green) that is localized both to the cell body and dendrites (MAP2, red) in WT mice (female data shown). *P < 0.05; **P < 0.01, ***P < 0.001, unpaired 2-tailed *t* tests. All data represent the mean ± SEM.

In the Hb of RIIα-KO mice, cAMP-stimulated PKA enzymatic activity was significantly decreased and basal activity tended to be blunted (Figure 2A). However, PKA enzymatic activity was unchanged in prefrontal cortex and striatum, 2 regions that provide direct input to the Hb (Figure 2A). Thus, disrupted cAMP signaling in the MHb is due to cell-autonomous *Prkar2a* deficiency. Further, the impact on cAMP-stimulated PKA activity suggests a blunted response to upstream signaling events in response to stimuli and not just a generalized decrease in activation under basal conditions.

Because cells in the vMHb release acetylcholine onto the IPN, the primary efferent of the MHb, we measured acetylcholine levels in the IPN. The Hb highly expresses nAChR, subtypes α3, β3, and β4; therefore, habenular acetylcholine concentrations were also measured. Acetylcholine concentrations were significantly lower in both the Hb and IPN of RIIα-KO compared with WT mice (Figure 2B). Additionally, we found comparable levels of glutamate in Hb between genotypes and decreased glutamate concentrations in the IPN in RIIα-KO mice (Figure 2C). To investigate alterations in the PKA subunit expression in the Hb, we quantified the mRNA and protein levels of the PKA subunits known to compensate for perturbations in the PKA system. mRNA levels of *Prkar1a* and *Prkaca* did not differ in RIIα-KO Hb compared with WT mice, but RIα protein tended to be lower and Cα was significantly reduced in RIIα-KO Hb lysates (Figure 2, D and E). Total PKA catalytic subunit (α, β, and γ) protein levels were also lower in Hb of RIIα-KO mice (Figure 2E). This is not unexpected because PKA is typically not regulated at the transcriptional level but is instead regulated via posttranslational changes in the stability of the PKA holoenzyme or free catalytic subunit (29).

### Localization of PKA catalytic subunits to MHb dendrites is disrupted in RIIα-KO mice.

Cellular localization of the PKA catalytic subunits and RIIα was investigated in the MHb at the point where the dMHb and vMHb meet in the center of the structure. Altered subcellular localization of PKA subunits can affect neuronal signaling and influence cellular function and phenotypic characteristics (30). PKA catalytic subunits localized to both the cytoplasm and the nucleus within the cell body and to the dendrites in the MHb of WT mice (Figure 2F, left panel). In RIIα-KO mice, however, localization of PKA catalytic subunits in dendrites was severely impaired, and total catalytic subunit expression appeared decreased, a feature that was supported quantitatively by Western blot (Figure 2F, right panel; Figure 2E). In WT mice, PKA RIIα was localized to the cytoplasm as well as to dendrites in MHb neurons, which was absent in RIIα-KO mice (Figure 2G). MHb excitatory inputs come through the stria medullaris from the septum, the nucleus accumbens (NAc) and the cholinergic broca diagonal band (3, 31, 32), and high expression of GABA-B receptors in the MHb (33, 34) suggests strong inhibitory inputs. The diverse connectivity of the MHb demonstrates a clear linkage to rewards circuitry involving both inhibitory and excitatory inputs that could be disrupted by impaired postsynaptic signaling due to PKA deficiency in the MHb.

### RIIα-KO mice have decreased intake of palatable food when provided chronic ad libitum access.

**Figure 3:**
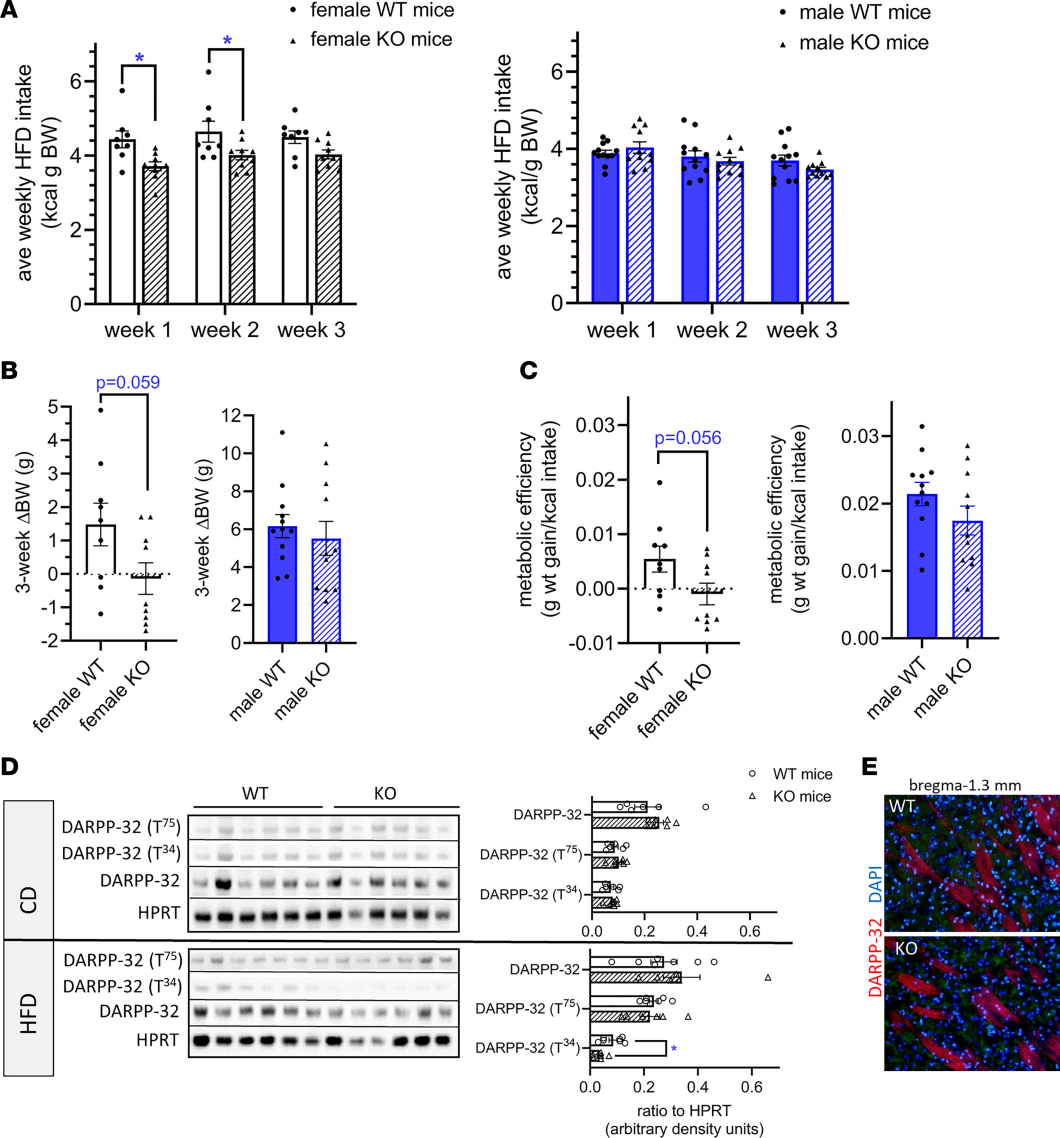
RIIα-KO mice had decreased HFD intake, sucrose solution intake, and sucrose preference compared with WT mice and differences were influenced by sex. (**A**) Average weekly intake of HFD during a 3-week period of ad libitum access was adjusted for body weight; *n*= 8–12/sex/genotype; repeated-measure 2-way ANOVA with multiple comparisons (Bonferroni’s post hoc test). (**B**) Difference in body weight (ΔBW) from outset of HFD intake experiment until the end of week 3 (1 negative data point for MKO not shown on graph); *n* = 8–12/sex/genotype; 2-way unpaired *t* test; *n*= 8–12/sex/genotype, and (**C**) metabolic efficiency (kcal intake/wt gain) for female and male RIIα-KO and WT mice during 3-week HFD-feeding period (1 negative data point for FKO not shown on graph); *n* = 9–12/sex/group, 2-way unpaired *t* test. (**D**) Representative Western blot of striatal lysates from mice fed CD (top) or HFD (3 weeks) (bottom) for DARPP-32 and DARPP-32 (T^34^) with HPRT as the loading control and densitometry analysis; 2-way unpaired *t* test, female data shown. (**E**) Representative IF image of post-HFD (3 weeks) striatum probed for DARPP-32 and counterstained with DAPI (*n* = 3 mice/genotype, females). Original magnification, 20×. All data are mean ± SEM; *P < 0.05. MKO, male RIIα-KO; FKO, female RIIα-KO.

To determine whether RIIα-KO mice consume less palatable HFD when given free access, we provided young adult male and female RIIα-KO and WT littermate mice ad libitum HFD for 3 weeks. Female RIIα-KO mice consumed less energy than their WT littermates (*P* = 0.0091, 2-way ANOVA), a phenomenon not observed in male mice (Figure 3A). Differences between genotypes were greater before adjusting for body weight (data not shown). Binge eating affects both sexes, but occurs more often in women (35), a sex difference that has been replicated in rodent models (36). Studies with dopamine (DA) antagonists have demonstrated that DA is important for the learned responses to food that relate directly to food reward reinforcement (37, 38). Removal of HFD after chronic exposure drives dysregulation of DA signaling in female, but not male, mice. Interestingly, when exposed to HFD for longer periods, a pattern of markedly reduced intake emerged in male RIIα-KO mice (data not shown). Cumulative energy intake for the 3-week study was markedly higher in female WT compared with KO mice (WT: 13.50 ± 0.51 kcal/g BW and KO: 11.76 ± 0.33 kcal/g BW) and did not differ between males (data not shown). Female RIIα-KO mice tended to gain less weight during HFD feeding (*P* = 0.059) (Figure 3B), and metabolic efficiency did not differ significantly from WT littermates (Figure 3C), consistent with our previous indirect calorimetry studies that showed only a small difference in resting volume of O_2_ during HFD, but not chow diet (CD), feeding (23).

Palatable foods are naturally rewarding and their consumption leads to the acute striatal DA release (39). Although striatal PKA enzymatic activity was not altered in RIIα-KO mice on a normal chow diet in vitro, HFD increases striatal PKA signaling and alters phosphorylation of DA- and cAMP-regulated phosphoprotein-32 (DARPP-32) in mice (40). DARPP-32 is a key integrator of DA and glutamate signaling in the basal ganglia that is highly expressed in striatal spiny neurons and can act either as an inhibitor of protein phosphatase 1 or PKA via T^34^ or T^75^ phosphorylation, respectively (41). Based on this and habenular complex connectivity in which the MHb is a central mediator of DA signaling between the NAc and ventral tegmental area (VTA) (3), we investigated striatal phosphorylation of DARPP-32. We found significant alterations (*P* = 0.02) in DARPP-32 phosphorylation after chronic HFD, but not CD, feeding in KO compared with WT mice. Phosphorylation of DARPP-32 at T^34^ was decreased in mutant mice after HFD but not CD feeding compared with WT littermates (Figure 3D; top: CD, bottom: HFD). Phosphorylation of DARPP-32 at T^75^ was unchanged in RIIα-KO mice irrespective of diet (Figure 3D). Striatal sections from WT and RIIα-KO mice stained for DARPP-32 did not appear to have different cellular distribution of DARPP-32 (Figure 3E). Decreased striatal DARPP-32 has been reported in the ΔFosB mouse, a model of increased reward sensitivity characterized by decreased HFD intake and lower levels of striatal phosphorylated cAMP-response element binding protein (Ser^133^) (42). Our results suggest that blunted striatal DARPP-32 T^34^ phosphorylation is associated with decreased intake of HFD in RIIα-KO mice. Rodent studies have shown that chronic HFD causes changes in the DA signaling system, including lower basal DA levels in the NAc (39, 43). Furthermore, acute or chronic treatment with the selective serotonin reuptake inhibitor (SSRI) fluoxetine leads to increased phosphorylation of DARPP-32 at T^34^ and decreased phosphorylation at T^75^ in the striatum (44). Here we show differences in striatal DARPP-32 phosphorylation in RIIα-KO mice after 3-week HFD feeding and decreased intake among females and males carrying a mutation (over a longer period of HFD exposure).

### Fasted RIIA-KO mice have decreased drive for food reward.

**Figure 4:**
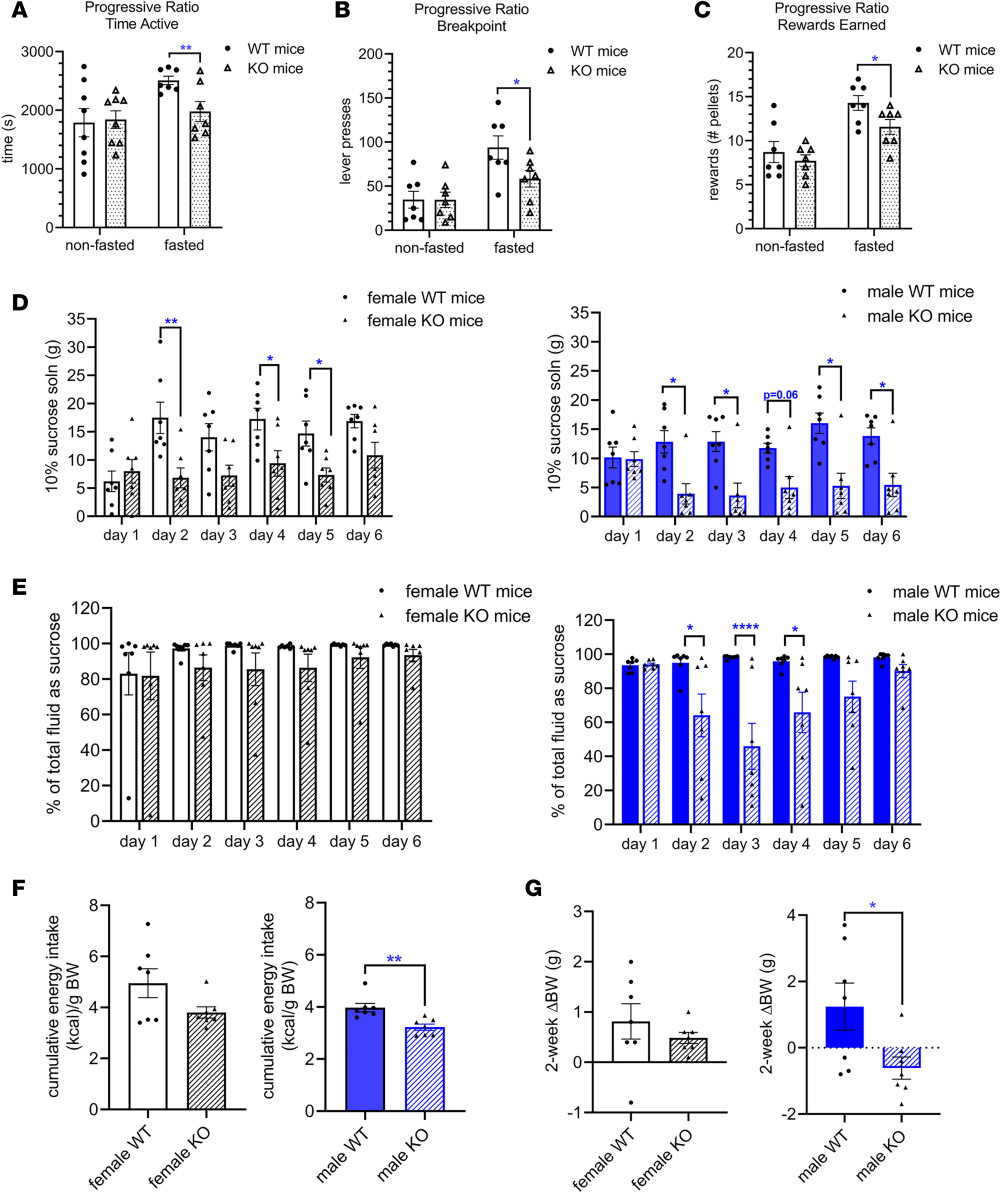
RIIα-KO mice had decreased motivation for food rewards, sucrose solution intake, and sucrose preference compared with WT mice; differences were influenced by sex. (**A**) Amount of time spent engaged in a progressive ratio (PR) operant task, (**B**) breakpoint, and (**C**) number of food pellets earned by WT and RIIα-KO littermates during PR operant task in fed and fasted states; *n*= 7–8/group, (female mice), analyzed by unpaired 2-way *t* tests. (**D**) Average daily intake of 10% sucrose solution of female and male RIIα-KO and WT mice; repeated-measure 2-way ANOVA with multiple comparisons (Bonferroni’s post hoc test) and (**E**) sucrose preference, measured as (daily sucrose solution intake [g]/total fluid intake [g]) × 100; sucrose intake and preference analyzed by repeated-measure 2-way ANOVA with multiple comparisons (Bonferroni’s post hoc test). (**F**) Cumulative total energy intake adjusted for body weight for female and male mice during 2-week experiment, measured as (sucrose kcal + chow kcal)/g BW, analyzed by 2-way unpaired *t* test (male) and Mann-Whitney nonparametric rank test (female). (**G**) Change in body weight during the 2-week sucrose intake experiment, analyzed by 2-way unpaired *t* test (male) and Mann-Whitney (female). For sucrose studies, *n* = 9–12/sex/group. All data are mean ± SEM; *P < 0.05; **P < 0.01, ****P < 0.0001.

RIIα-KO and WT littermates trained to perform an operant lever press task on a fixed ratio schedule of food pellet delivery were then subjected to a progressive ratio operant task in both the fed and fasted states. There were no differences in learning the operant task as assessed by percentage of correct and incorrect lever presses and in achieving the goal of earning 50 food pellets with at least 80% correct lever presses (data not shown). Additionally, the amount of time spent engaged in the operant task and the drive to work for food reward assessed (i.e., breakpoint) in nonfasted mice did not differ between genotypes (Figure 4, A–C). After a 14-hour overnight fast, RIIα-KO mice spent less time engaged in the PR task (Figure 4A), had a lower breakpoint, and earned fewer food reward pellets than their WT littermates (Figure 4, B and C).

### RIIA-KO mice drink less sucrose solution and have blunted sucrose preference.

To further evaluate the role of *Prkar2a* in hedonic drive for natural rewards, we performed sucrose preference tests in male and female mice using 10% (wt/vol) sucrose solution and a standard 2-bottle paradigm. Sucrose preference is a well-established test for anhedonia, defined as the lack of ability to experience pleasure from rewarding or enjoyable activities (45). When mice were given 24-hour access to both 10% sucrose and water for 3 consecutive days per week over a 2-week period, multiple comparisons revealed similar sucrose intakes between WT and RIIα-KO mice on day 1, but RIIα-KO mice had lower intake levels than WT mice on subsequent days (Figure 4D). There was a significant genotype effect on sucrose intake for female and male mice (*P* = 0.0095, *P* = 0.0053, 2-way ANOVA). Similarly, after the initial 24-hour period, sucrose preference was significantly decreased in male RIIα-KO mice (*P* = 0.0073), highlighting a sex difference in the *Prkar2a*-mediated regulation of sucrose reward (Figure 4E).

Despite the lack of differences in energy intake from chow between genotypes (data not shown), male RIIα-KO mice had decreased total energy intake due to differences in sucrose consumption and gained less weight than WT littermates during the 2-week experiment (Figure 4, F and G). There were no differences in cumulative total energy intake or weight gain after sucrose access in female mice (Figure 4, F and G).

Circuits between the lateral hypothalamus and the VTA, an indirect target of the MHb, play a role in sucrose seeking and reward encoding, (46) and tachykinins (derived from the MHb) are involved in umami and perhaps other taste modalities (47). VTA glutamate neurons have also been associated with positive reinforcement during reward-based operant tasks mediated by their release of GABA (48). Thus, disruption of these signals through decreased MHb signaling to the VTA via the IPN may act to impair reward processing and reinforcement.

### RIIA-KO mice have increased drive for voluntary running.

**Figure 5:**
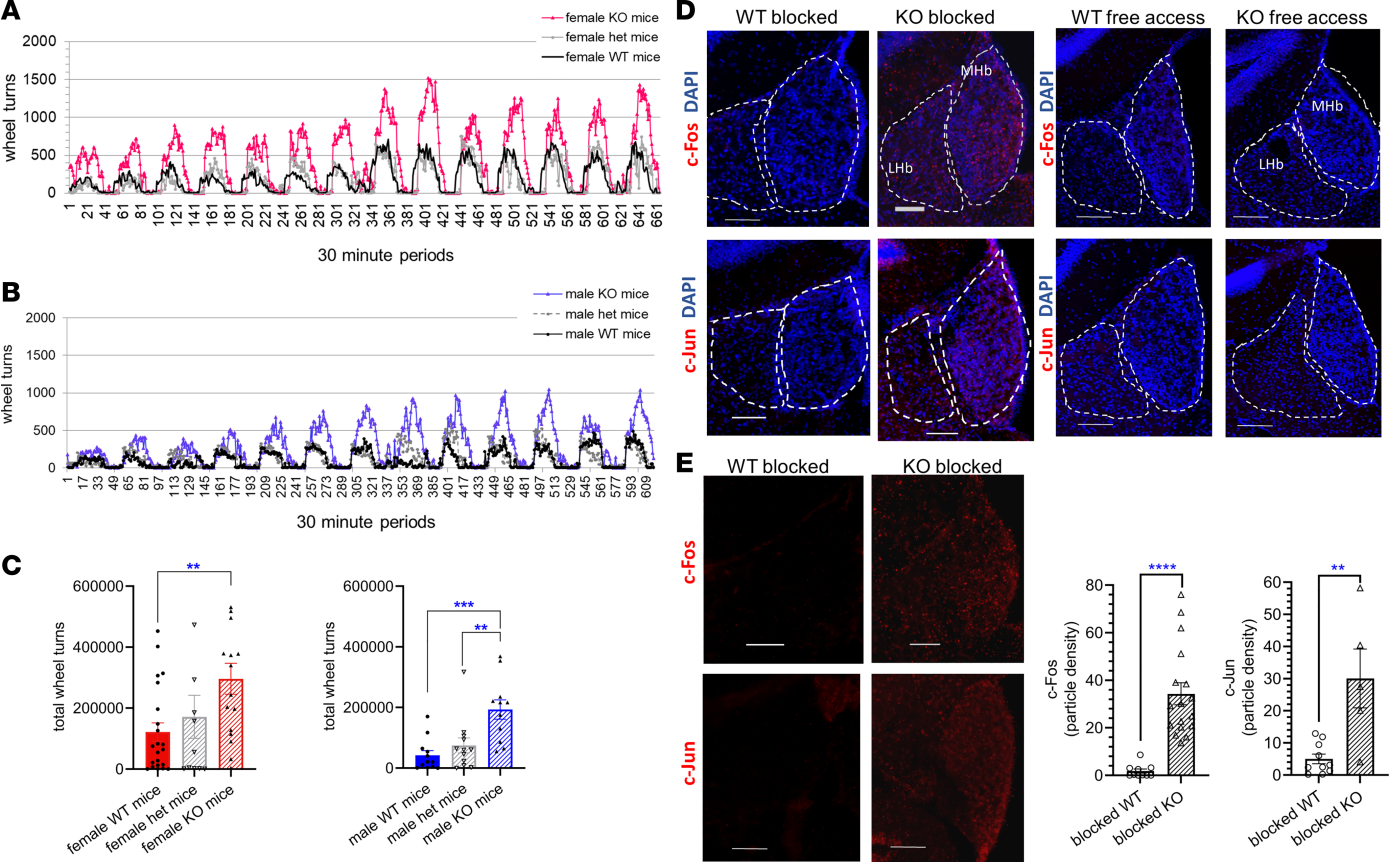
RIIα-KO mice run more than twice the distance of their WT littermates during home cage running wheel access. Heterozygosity for *Prkar2a* rescued the running phenotype. Voluntary running activity was graphed in bins of 30 minutes over a 2-week period for (**A**) female (*n* = 11–24/genotype) and (**B**) male (*n* = 8–12/genotype) RIIα-KO, RIIα^+/–^, and WT mice and (**C**) the total number of wheel turns during the 2-week period, analyzed by 1-way ANOVA with multiple comparisons (Bonferroni’s post hoc test). (**D**) Representative staining for c-Fos or c-Jun (red) (merged with DAPI) in WT and RIIα-KO mice either permitted to run as already acclimated for 2 weeks prior or blocked from running at the outset of the dark cycle (1800 hours). (**E**) The same images (4D) are shown with only c-Fos or c-Jun (red) and particle density of c-Fos and c-Jun was analyzed with ImageJ software (NIH); unpaired 2-way *t* tests, *n* = 5–17 sections from a total 3–5 mice per IEG/genotype (3 males, 7 females). Particle density is defined as the number of counted particles divided by the area ratio of the MHb, with the latter being the area of the MHb over the total image area. All data represent mean ± SEM; scale bars represent 100 μm; **P < 0.01, ***P < 0.001, ****P < 0.0001.

Motivation for exercise can play an important role in energy homeostasis, and this drive is regulated by shared circuitry that regulates other natural rewards yet has distinctions from those mediating food reward (i.e., taste vs. locomotor activity). The Hb plays a role in regulating the temporal pattern of locomotor activity throughout the night (49). Moreover, both developmental ablation of dMHb neurons, and maturation defects in the MHb, severely impair voluntary wheel running behavior in mice (19). Thus, we investigated voluntary exercise performance of RIIα-KO mice. When provided with home cage running wheels, RIIα-KO mice ran 2–3 times the distance of their WT littermates (Figure 5, A and B). *Prkar2a* heterozygosity rescued the high running phenotype of RIIα-KO mice to levels of WT mice. These data suggest that even partial restoration of RIIα-mediated PKA activity and holoenzyme localization suffices to reverse the change in PKA signaling responsible for the observed motivation for running. Total wheel turns for the 2-week running experiment was significantly lower for both WT and RIIα^+/–^ mice compared with RIIα-KO mice for both sexes (Figure 5C). The timing of running activity across light/dark cycles and within the active dark cycle was as expected with spikes of activity in the early dark period and tapered activity in the later part of the dark cycle and did not differ among genotypes. We previously showed that normal home cage locomotor activity was not different between WT and RIIα-KO mice of either sex (23). Denial of the expected access to running wheels was identified as activating the striatum, lateral hypothalamus, and frontal cortex in mice selectively bred for running, suggesting that omission of reward led to anxiety or stress (50).

Given the increased motivation for running in RIIα-KO mice, we hypothesized that mice carrying a mutation and accustomed to daily wheel running might experience stress that could be detected by Hb activation in the absence of this natural reward. The MHb was identified as 1 of 6 limbic regions that are susceptible to stress-induced c-Fos expression (51), and c-Fos was identified in MHb and LHb in response to restraint stress and forced swim test (52). Therefore, we decided to assess both c-Fos and c-Jun expression in response to being blocked from the expected running wheel access. In RIIα-KO mice, blocking access to running wheels before onset of the dark cycle resulted in increased c-Fos and c-Jun expression in the MHb (Figure 5, D and E). Induction of c-Fos and c-Jun was not observed in the MHb of WT mice that were blocked from running. Regardless of genotype, no immediate early gene (IEG) induction in the Hb was observed in mice that had continued access to running wheels (Figure 5D, right panels), suggesting that hyperactivation of the MHb may be associated with stress or anxiety related to withholding the pleasurable experience of running. Additionally, when similar studies were conducted after mice were habituated to sucrose access, no IEG induction was observed in WT or KO mice under either reward or blocked reward conditions (data not shown).

### Recombinant adeno-associated viral vector–mediated habenular Prkar2a reexpression rescues sucrose preference and running phenotypes.

**Figure 6:**
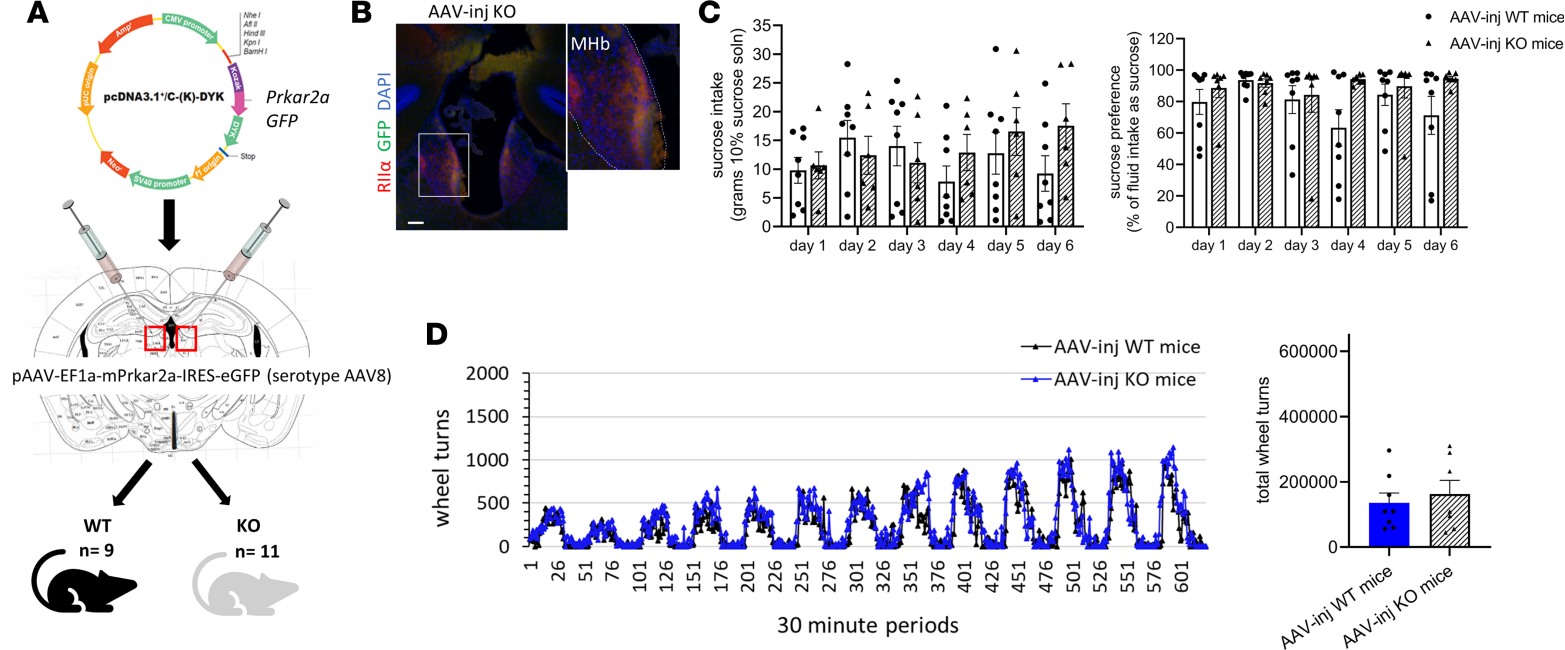
Stereotaxic rAAV-mediated reexpression of *Prkar2a* rescued the sucrose and running phenotypes of RIIα-KO mice. (**A**) Map of *Prkar2a* expression vector used to generate injectable rAAV and diagram of the injection strategy. (**B**) Representative image of coronal section from injected RIIα-KO mouse of a successful rAAV injection with expression of RIIα and GFP in MHb with magnified portion on right; scale bar represents 100 μm; for magnified portion, original magnification, 40×. (**C**) Sucrose intake and sucrose preference tests for rAAV-injected WT and KO mice revealed no differences between genotypes (*n* = 6–8/group; 7 males, 7 females), and (**D**) 2-week running wheel activity of rAAV-injected WT and KO mice did not differ between genotypes; (*n* = 7–8/group; 6 males, 9 females), repeated measure 2-way ANOVA with multiple comparisons (Bonferroni’s post hoc test) and a 2-tailed *t* test were used for sucrose and running analyses, respectively; all data represent the mean ± SEM.

We hypothesized that the behavioral phenotypes observed were driven specifically by *Prkar2a* deletion in the Hb. We delivered a recombinant adeno-associated viral vector (rAAV) with a construct containing *Prkar2a* and GFP via bilateral stereotaxic injections to young adult WT and RIIα-KO mice (both sexes) (Figure 6A). Pilot injections first with retrobeads and then with a GFP-containing rAAV were used to confirm injection coordinates. Postexperimental injection accuracy was confirmed by immunofluorescence for each mouse (Supplemental Figure 2; Figure 6B is a representative image of the expected reexpression of PKA RIIα). RIIα-KO mice with off-target injections were classified by lack of immunofluorescent signal for RIIα and GFP in the MHb and subsequently excluded from the data analysis. Two to 3 weeks after a surgery, a period that was sufficient for recovery, and to ensure adequate RIIα protein expression, sucrose preference tests were performed followed by a 2-week washout period before initiating 2-week running wheel experiments. Reexpression of *Prkar2a* in the MHb rescued the sucrose intake and preference phenotype of RIIα-KO mice (Figure 6C) as well as the increased voluntary running phenotype (Figure 6D). There were no differences between mean sucrose intake or preference levels or total wheel turns in the rAAV-injected WT or rAAV-injected RIIα-KO mice, confirming that habenular *Prkar2a* inversely regulates voluntary exercise and sweet reward responses.

## Discussion

Across species, a positive response to natural rewards is an innate survival mechanism that is driven by the cognitive processing of pleasure experienced from activities like eating, running (as prey or predator), or sex. While being able to experience the rewarding aspects of food is evolutionarily vital, overriding satiety signals in favor of the overconsumption of high-fat and sweet foods can lead to obesity, metabolic dysregulation, and other related comorbidities. Achieving weight loss and maintaining energy balance by moderating food intake and increasing physical activity underlies the battle against dietary obesity and weight gain. The Hb is central to reward and aversion systems, which are both necessary for maintaining balance in processing reward stimuli. The LHb is critical in transmitting negative-reward signals, and rewarding stimuli cause decreased LHb activity in concert with increased DA activity, while the reverse is true of aversive stimuli (2, 53). Although the Hb has been hypothesized to serve as a nexus of the complex reward circuitry with a key role in maintaining the balance between reward-seeking and avoidance behaviors (8), much less is known about the roles for the MHb in these processes.

Here we identify an unexpected role for PKA RIIα in the MHb in the simultaneous positive regulation of food rewards and negative effect on the drive to exercise, behaviors that were both reversed with RIIα deficiency. The diminishment of both drives is characteristic of the anhedonia observed in major depressive disorder (MDD) (54), and LHb hyperactivation has been associated with both the neurobiologic dysregulation and the motivational symptoms of depression (55). We demonstrate a significant decrease (*P* = 0.0001)in cAMP-stimulated PKA activity as well as altered dendritic localization of “active,” free PKA catalytic subunits in the MHb of the RIIα-KO mouse. Given the direct and unidirectional input from the MHb to LHb (13), it seems likely that input to the LHb is likely also affected by the altered MHb PKA signaling in RIIα-KO mice. Decreased LHb activity inversely affects local DA activity that has downstream effects on VTA, a pathway that has notable overlap with the VTA-lateral hypothalamus-NAc pathway. Impaired DA signaling is a common thread that connects compulsive behaviors related to food intake and substance abuse and the motivation symptoms of depressive disorders. In both obesity and substance abuse, the dysregulation of DA signaling and subsequent changes in reward circuitry can fuel the cycle of compulsive drug or compulsive food consumption.

Altered DA signaling downstream of MHb after chronic HFD exposure was evidenced in the RIIα-KO mouse by decreased striatal DARPP-32 T^34^ phosphorylation (Figure 3D). In intact neurons, T^34^ phosphorylation inhibits protein phosphatase 1, which in turn inhibits D_1_ DA signaling (41). After roux-en-Y gastric bypass, mice had increased DA D1R activity and reduced fat intake via PPARα-vagal-D1R signaling, unlike sham-operated mice (56). Additionally, studies in the ΔFos-overexpressing mouse, a model of increased reward sensitivity, confirm the importance of NAc feedback to the VTA in regulating DA signaling and moderating HFD intake. While chronic HFD led to decreased mRNA expression of tyrosine hydroxylase and DA transporter in the VTA of control mice, levels of both were increased in the reward-sensitive ΔFos mouse (42).

Blunted sucrose intake was a clear phenotypic characteristic of mice lacking *Prkar2a* and was strongest in males (Figure 4, D and E). Whereas sucrose intake escalated in WT mice after its introduction and a high level of daily sucrose intake was maintained, the intake pattern for RIIα-KO mice suggests decreased motivation and altered reward processing. It is important to note that RIIα-KO mice prefer both HFD and sucrose solution when offered the choice between normal chow or HFD and 10% sucrose or water, respectively. The significant decrease in breakpoint, time engaged in the task, and number of rewards earned during operant progressive ratio tasks in fasted RIIα-KO compared with WT mice further suggests the presence of an intact reward circuit but decreased appetitive motivation that could lead to the “moderation” phenotype seen in RIIα-KO mice with respect to palatable foods.

Sucrose preference and the behavioral and physiologic responses to chronic HFD exposure were sex-dependent in RIIα-KO mice. Our observations with regards to sex differences in the recorded behaviors and metabolic parameters are consistent with other mouse models of PKA deficiency or overexpression, in which sexual dimorphism is a common characteristic (57). While the requirement to include females in clinical and basic research protocols is relatively recent, mounting evidence suggests key differences in the regulation of behaviors linked to DA (and other monoamine) signaling. We report a more pronounced decrease in sucrose intake and preference in male compared with female RIIα-KO mice, while decreases in intake, weight gain, and metabolic efficiency during chronic HFD feeding were significant only in female KO mice. Clinical studies of depressive disorders reinforce key differences in the prevalence of depression and the response to antidepressant drugs between sexes (58). Binge eating is more frequent in women (35), a finding that is replicated in rodents (36). Thus the inhibition of HFD intake in female mice via *Prkar2a* deletion is particularly interesting. Additionally, depression is more prevalent in women (59), who are also significantly more sensitive to the antidepressive effects of ketamine (60) and traditional SSRI antidepression drugs via an ovarian hormone–dependent mechanism (60). In MDD, women experience greater anxiety and somatic symptoms that include disturbances in sleep, appetite, and pain (61). Sex-dependent midbrain monoamine regulation likely underlies a number of signaling processes involved in mood, intake of palatable foods, and the pleasure derived from naturally rewarding behaviors including those observed in the RIIα-KO mouse. Further interrogation of behavior and physiologic responses to both rewarding and aversive stimuli in male and female RIIα-KO mice may provide deeper insight into the mechanisms that underlie the observed differences. Our data show that comparable reductions in Hb PKA activity and neurotransmitter levels in the Hb and IPN in male and female mice elicit distinct effects in reward signals that can affect intake and body weight.

The dysregulated PKA signaling induced via *Prkar2a* deletion in the MHb causes decreased acetylcholine release in both the Hb and the IPN, as well as decreased glutamate levels in the IPN (Figure 2, B and C). Hb acetylcholine signaling has been linked to both nicotine addiction and the aversive aspects of withdrawal (62) and to depressive disorders (63), and more recently nicotine addiction has been linked to impaired glucose control via Hb-gut connections (5). Although downregulation of cholinergic signaling causes anhedonia-like behavior but not despair (63), the developmental ablation of MHb neurons using a *Pou4f*-Cre driver in mice blunts both voluntary exercise and sucrose drinking, without other depression-related symptoms (19). Here we show that impaired *Prkar2a* regulation of PKA activity in MHb clearly enhanced the drive for voluntary exercise that could occur by altered localization of active PKA or decreased total activity. However, the previously described mouse models and disease states, such as MDD and other depressive disorders, obesity, and the first 2 of the 3 stages of the drug addiction cycle (intake/binge, aversive cycles) (64), involve the regulation of these drives in the same direction. *Prkar2a* was expressed in heterogenous cell populations within the dMHb and vMHb, in both substance P– and acetylcholine-expressing cells, and was expressed in glia and neurons, which likely explains the differential regulation of exercise and food reward drives. We have shown the direct impact of *Prkar2a* deletion on acetylcholine and glutamate levels. *Prkar2a* is also highly expressed in *Tac1-* and *Tac2-*expressing cells of the MHb, and thus, further study of PKA regulation in the various distinct cell subtypes is warranted.

Apart from what was learned from studies in the ΔFos-overexpression and *Pou4f*-Cre–driven MHb neuronal ablation mouse models, the RIIα-KO mouse implicates the downregulation of PKA activity in MHb in the regulation of 2 overlapping but distinct pathways. Reexpression of *Prkar2a* in the Hb of the global RIIα-KO mouse rescued the sucrose and running phenotypes, confirming that both behaviors are mediated by changes in habenular PKA signaling. Whereas the observed blunted response to sucrose and HFD and the decreased appetitive drive to obtain food rewards in the fasted state resemble anhedonia-like behaviors associated with MDD, the decreased locomotor activity typically observed with anhedonia was absent in this mouse model. While the connections to food reward behaviors in the RIIα-KO mouse are clearer, the link to enhanced voluntary activity seems more complicated despite the known relationship between reward seeking and locomotor sensitization (65). The inverse regulation of consummatory drive and the drive to engage in exercise caused by *Prkar2a* deficiency generates a desirable phenotype of sucrose and HFD intake moderation and of enhanced motivation for exercise. We identify habenular *Prkar2a* as a new player in regulating the habenular complex (aka, dorsal diencephalic conduction system) and provide new insights into the role of habenular PKA signaling, the regulation of hedonic drive, and susceptibility to dietary obesity.

## Methods

### Mice.

RIIα-KO mice were obtained from Mutant Mouse Resource and Research Centers and have previously been described (22). RIIα heterozygous breeding pairs were bred on a C57BL/6 background to generate WT and KO littermates. This mouse line has been bred in our mouse facilities for approximately 10 years, which ensures more than 99% C57BL/6 background. A standard 0600 hours/1800 hours light/dark cycle was consistently maintained with an average temperature of 73°F. Mice were all handled regularly by the same individuals for the at least 2–3 weeks leading up the behavioral studies.

### Ad libitum HFD feeding studies.

To measure intake of palatable chow, we used Bio-Serv F3282, a soft HFD that provides 5.49 kcal/g and derives approximately 15%, 59%, and 26% of total energy from protein, fat, and carbohydrate, respectively. Young adult (12- to 16-week-old) male and female WT and RIIα-KO littermates were individually housed and provided free access to drinking water and HFD. Body weight and weight of the food consumed were measured weekly for 3 weeks. Mice were maintained on the same 12-hour light/12-hour dark cycle and temperature and humidity conditions that they had been acclimated to from birth.

### Sucrose intake and sucrose preference test.

Sucrose preference was evaluated in individually housed WT and RIIα-KO littermates by providing identical bottles containing water and 10% (wt/vol) sucrose solution side by side daily for 3 consecutive days a week for 2 weeks. The positions of sucrose and water were alternated daily, and the amounts of sucrose solution and water consumed were determined by weighing each bottle when removed from the cage, and for sucrose solution, before replacement with fresh solution. Absolute sucrose solution, water, and control chow (CD) (NIH-31) intakes were analyzed, and sucrose preference was calculated as a percentage: (preference = sucrose solution intake [g])/(water intake [g] + sucrose solution intake [g]) × 100.

### Operant conditioning positive reinforcement studies.

Young adult mice (3–6 months old) were individually housed in cages with a divider and calorie restricted for 1 week to achieve 90% of initial body weight and maintained on an 85% calorie-restricted diet for the initial conditioning phase. Briefly, 85% of ad libitum intake was determined based on the average daily intake for 3 days. Once 90% of starting body weight was achieved, body weight was monitored daily, and caloric restriction continued with adjustments made as needed to ensure optimal weight throughout the training phase. Purified Rodent Dustless Precision Pellets (14 mg, Bio-Serv) were used as food rewards.

Mice were randomly assigned to either right or left lever press and were trained with a fixed ratio 1 (FR1) schedule in which 1 food pellet was delivered for each correct lever press. The criteria for successful completion of the FR1 and FR5 tasks were receiving 50 food pellets with at least 80% correct lever presses within the 60-minute test period. Upon successful completion of the FR1 task for 2 consecutive days, mice progressed to the FR5 schedule and then to the PR. Ad libitum feeding was reintroduced for FR5 and PR portions of the study.

### Voluntary running behavior and blocked running wheel experiments.

Individually housed young adult mice (3–6 months old) were provided home cage running wheels for 2 weeks (Med Associates Inc). Daily running and total wheel turns were analyzed as 30-minute bins for the entire 2-week period. To test the effects of blocking the anticipated natural reward of running on IEG expression in the Hb, we used mice that had been provided free access to home cage running wheels for 2 weeks prior (*n* = 4–6/group). Running wheels were locked but left inside of home cages 2 hours before the onset of the dark cycle (1600 hours). Between 1.5 and 2.5 hours after dark cycle onset, when running levels are typically high (1930 to 2030 hours), mice were transcardially perfused and brains harvested and processed as later described for immunofluorescence staining for c-Fos and c-Jun.

### Dissection of brains for PKA enzymatic activity, ELISA, and Western blot.

Brains were cut into sections that were 150–200 μm thick and kept cold while dissecting using a microscope (SMZ 1500, Nikon). Prefrontal cortex, striatum, and Hb were dissected based on stereotaxic coordinates using the following landmarks: prefrontal cortex (bregma 3.0 to –2.5 mm), bilateral samples taken just above and excluding the orbital area; striatum (bregma 1.5 to –0.2 mm), bilateral samples below and on the interior side of the genu of the corpus callosum and along the exterior edge of the lateral ventricle; Hb (bregma –1.0 to –2.0 mm), bilateral samples taken directly adjacent to third ventricle just below the stria medullaris and above the paraventricular nucleus of the thalamus; IPN (bregma –3.35 to –3.45 mm) was taken from either side of the midline just dorsal to the middle cerebellar peduncle and ventral to the ventral tegmental decussation, based on *The Mouse Brain in Stereotaxic Coordinates* (Franklin and Paxinos) (66). Dissected samples were immediately snap-frozen in liquid nitrogen and stored at –80°C until assay.

### PKA enzymatic activity assay.

Tissues were homogenized in freshly prepared lysis buffer (10 mM Tris-HCl pH 7.5, 1 mM EDTA, and 1 mM dithiothreitol with 0.5 mM PMSF and protease inhibitor cocktail I, 1:100; EMD Biosciences). BCA assays were performed as per manufacturer’s protocol to determine the total protein concentrations of samples (Pierce, Thermo Fisher Scientific). Samples were diluted to 1 μg/μL and 10 μL of total protein was used for each reaction. PKA enzymatic assays were performed by kemptide assay, using 25 μM kemptide (Leu-Arg-Arg-Ala-Ser-Leu-Gly), as previously described with and without cAMP (5 μM) (67). All reactions for basal and cAMP-stimulated (total) PKA activity were carried out in duplicate. Additionally, activity values for replicate reactions that were incubated in the presence of protein kinase inhibitor (5 nM) were subtracted from activity values to account for nonspecific kinase activity.

### Quantification of acetylcholine.

IPN acetylcholine concentrations were determined by choline/acetylcholine assay kit (Abcam, catalog ab65345) as per the manufacturer’s protocol. Samples were weighed before homogenization to standardize the total amount of tissue analyzed. Tissues had been previously snap-frozen after microdissection from 150 to 200 μm thick sections at bregma –3.45 to –3.5 mm as previously described. After snap-freezing, samples were stored at –80°C.

### Western blotting.

Habenular and striatal lysates were prepared as described for PKA enzymatic activity assays. Per lane, 10 μg of total protein was loaded onto 4%–12% Bis-Tris gels (Bolt Plus, Invitrogen, Thermo Fisher Scientific) and run for 35 minutes at 165 V. For each gel, 7 μL of WesternSure prestained protein ladder was loaded onto a separate lane (LI-COR Biosciences). Protein was transferred onto nitrocellulose membranes using a semidry apparatus for 30 minutes (TransBlot Turbo, Bio-Rad), stained with Ponceau S stain, washed with 1× TBS with 0.1% Tween-20 (1× TBST), and then blocked with 5% nonfat dry milk or bovine serum albumin in 1× TBST for 1 hour at room temperature. Membranes were then probed overnight with primary antibodies with gentle shaking at 4°C before washing 3 times with 1× TBST and probing for 1 hour at room temperature with the appropriate antibody and Precision Protein StrepTactin-HRP Conjugate (1:10,000, Bio-Rad). All Western blots were visualized using Pierce enzyme chemiluminescent substrate (Thermo Fisher Scientific) and a ChemiDoc analyzer (Bio-Rad). See Supplemental Table 1 for antibodies used, sources, and dilutions used.

### RNA extraction and relative mRNA expression analysis.

RNA was extracted from previously snap-frozen Hbs that had been stored at –80°C. RNA was extracted by adding 500 μL TRIzol (Invitrogen, Thermo Fisher Scientific) to each sample in a microcentrifuge tube preloaded with RNase-free beads (Next Advance) and homogenized using a Bead Ruptor Elite (Omni International) for 2 cycles, 20 seconds each, at a speed of 2.4. Samples were centrifuged for 5 minutes at 12,000*g* at 4°C. Supernatant was transferred to a clean tube for each sample and incubated for 5 minutes before adding 100 μL of chloroform, shaking by hand for 30 seconds, and incubating for an additional 3 minutes. Samples were centrifuged for 15 minutes at 12,000*g* at 4°C. The aqueous phase was carefully removed and RNA quantified by NanoDrop (Thermo Fisher Scientific). A total of 500 ng of RNA was used to make cDNA for each sample using SuperScript III First Strand Synthesis Supermix for Quantitative reverse transcriptase polymerase chain reaction (qRT-PCR) per the manufacturer’s protocol (Invitrogen, Thermo Fisher Scientific). qRT-PCR was performed on a VIIA7 instrument (Bio-Rad) using a standard 40-cycle program that included melt curve analysis. To determine relative mRNA expression levels of PKA subunits in Hbs, the following specific, preoptimized primers were used: *Prkar1a*: forward 5′-CGAAGAATCCTCATGGGAAG-3′, reverse 5′-CTCTCCTTGCACCACGATCT-3′; *Prkaca*: forward 5′-GAAAATCGTCTCTGGGAAGGT-3′, reverse 5′-TGGCAATCCAGTCAGTCGT-3; *Rplp0*: forward 5′-GAAAATCTCCAGAGGCACCA-3′, reverse 5′- ACCCTCCAGAAAGCGAGAGT-3′; *18S*: forward 5′-GCAATTATTCCCCATGAACG-3′, reverse 5′-GGCCTCACTAAACCATCCAA-3′. SYBR Green *Power* Master Mix (10 μL), 1 μL of cDNA, 8.4 μL H_2_O, and 0.3 μL of each primer (10 μM) were combined for each reaction well, and each reaction was performed in triplicate (Applied Biosystems, Thermo Fisher Scientific). Relative expression was determined using the 2^-ΔΔCt^ values that were calculated based on the average Ct values for the housekeeping genes determined to be optimal for the sample type (*18S* and *Rplp0*), and values were analyzed as the fold change from WT values (68). Melt curves and gel electrophoresis were used to confirm the expected PCR products.

### Immunofluorescence.

Mice were killed by slow replacement of air with CO_2_ (flow rate ≤ 5 L/min) followed by cervical dislocation. Mice were transcardially perfused with ice-cold 1× PBS (10 mL), followed by 4% paraformaldehyde (PFA) (20 mL). Whole brains were postfixed for 1 hour in 4% PFA, washed 3 times with 1× PBS, and then cryopreserved in 30% sucrose until brains sank. Brains were washed again 3 times with 1× PBS and snap-frozen in –80°C 2-methylbutane. Floating sections (35 μm) were blocked with 5% donkey serum in 1× PBS with 0.1% Triton X-100 and 0.1% glycine for 1 hour. Sections were incubated with primary antibodies overnight at 4°C with gentle shaking, then washed 3 times with 1× PBS with 0.1% Triton X-100 and then incubated with appropriate secondary antibodies for 1 hour at 4°C with gentle shaking. Sections were then washed 3 times with 1× PBS with 0.1% Triton X-100 and counterstained with DAPI (1:1000; MilliporeSigma). Antibodies were diluted in 2× diluted blocking buffer. See Supplemental Table 1 for antibodies and conditions used.

### RNA ISH.

Brains harvested from RIIα-KO and WT littermates were immediately snap-frozen in isopentane and stored at –80°C until cryosectioning. Brains were equilibrated at –20°C for at least 1 hours before being sectioned (20 μm thick) on a cryostat (Leica) and mounted directly onto Superfrost slides (Thermo Fisher Scientific). Slides were sealed in an airtight bag and stored at –80°C and then fixed in ice-cold 4% PFA for 1 hour at 4°C before use. After fixation, slides were dipped in ice-cold 1× PBS, then dehydrated in an ethanol gradient from 50% to 100% for 5 minutes at each step. Slides were stored in fresh 100% ethanol for up to 7 days at –20°C before hybridization. ISH was performed by first pretreating sections with an RNAscope protease inhibitor IV for 20 minutes and then probed using specific probes (Supplemental Table 2) and counterstained with DAPI following the manufacturer’s protocol (RNAscope, ACD Bioscience, Bio-Techne).

### Fluorescence microscopy.

For ISH expression studies, immunofluorescence experiments and cellular localization studies, images were captured using a confocal microscope with fluorescent filters while maintaining uniform exposure settings across samples within each experimental batch (Zeiss LS800). A magnification of either 20× (for cell counting and *Prkar2a* colocalization with markers) or 63× with oil (dendrite localization) was used.

### Quantification of colocalization of Prkar2a with MHb markers.

Colocalization analyses were done using the Zen blue software (Zeiss LS800). Sections were probed for *Prkar2a* and 1 to 2 other markers of MHb cell subtypes, including *Tac1*, *Tacr1*, *Tac2*, *Slc17a7*, and *Chat*, then counterstained with DAPI. Zen blue software was used to perform the particle and density analyses (Zeiss). The polygon drawing tool was used to outline the MHb as the area of interest. The colocalization tool was used as per the manufacturer’s recommended procedure to determine colocalization between *Prkar2a* and another marker and is widely used for such quantifications (69). Colocalization thresholds were kept constant within each brain section. The used data point was the colocalization coefficient 1: Rhoda-T3, which describes the ratio of colocalized points for *Prkar2a* and the targeted marker to total *Prkar2a* points in the selected area.

### c-Fos– and c-Jun–positive cell counting.

c-Fos– or c-Jun–stained brain sections from mice that were either blocked from or permitted to continue running as previously described were imaged with a confocal microscope using uniform settings (Zeiss LS800) and analyzed using ImageJ software (NIH). The color channels for each image were split apart. Analyses were conducted solely on the red channel image. An outline of the MHb was created using the polygon drawing tool. Image threshold was set to allow for analysis of only legitimate signal. Threshold settings were kept uniform throughout the analysis of each sample. The number of image particles was obtained using the Analyze Particles function. Particle density ratio was calculated by the equation: number of particles/area ratio of the MHb, the latter defined as the area of the MHb over the total image area. Area measurements were obtained using the Measure function.

### rAAV-mediated reexpression of Prkar2a.

The construct pAV-EF1a-mPrkar2a-IRES-EGFP was generated and packaged into an adeno-associated virus (serotype AAV8) (titer: 1.44 × 10^–13^ genome copies/mL; Vigene Bio; Figure 6). For construct validation, the vector was transfected into HEK293 cells (Transfectamine 2000, Invitrogen, Thermo Fisher Scientific), and cells were lysed at 48 hours posttransfection to confirm expression of *Prkar2a* and GFP gene products. Survival surgery was performed on male and female RIIα-KO and WT mice (*n* = 9–11/genotype) using aseptic techniques. Mice were anesthetized with 5% isoflurane and mounted on a stereotaxic frame with an integrated computer atlas (Leica Angle Two) to aid with injection angles. Topical lidocaine/prilocaine cream (2.5%/2.5%) and buprenorphine (0.1 mg/kg via subcutaneous injection) were administered for postoperative analgesia. Following midline incision, 1 mm holes were drilled bilaterally at the following injection sites: anterior/posterior: –1.53 mm from bregma, lateral: (–/+) 0.17 mm, depth: –2.5 mm. A glass micropipette was used to deliver a volume of 100 nL of rAAV at a flow rate of 0.1 μL/min at 10.08° angles. Retrobeads (Lumafluor) were used initially to confirm the injection coordinates. Mice were provided with topical lidocaine/antibiotic ointment and ketoprofen daily for at least 3 days following surgery and were observed regularly during the 2-week recovery period that also enabled expression of the target proteins before behavior testing. Upon completion of post–rAAV injection behavior testing, mice were sacrificed and perfused, and brains were prepared for immunofluorescence as described above. rAAV injections were validated by fluorescence microscopy targeted to the Hb and surrounding areas (Keyence) (Supplemental Figure 2). RIIα-KO and WT mice in which habenular expression of GFP and RIIα was not achieved were excluded from the experimental data.

### Statistics.

GraphPad Prism version 8.4.2 was used for all statistical analyses: α was set at 0.05 and a *P* value of less than 0.05 was considered significant. When applicable, 2-way unpaired *t* tests were used to compare WT and RIIα-KO mouse data when normality assumptions were met. For data that were not normally distributed, the Mann-Whitney rank test was used. For data sets including multiple time points, repeated measures 2-way ANOVA analysis was used to evaluate the effect of genotype and of genotype and time on intake levels and preference. The Geisser-Greenhouse correction was used as needed to correct for unequal variability of differences. Multiple comparisons for 1-way and 2-way ANOVA analyses were done using Bonferroni’s post hoc test to compare individual mean values for HFD intake, sucrose intake, and preference between genotypes. Two-way unpaired *t* tests were used for data that were normally distributed, and the Mann-Whitney rank test was applied to data that did not meet the normality assumptions. Tests used and *P* value ranges are detailed in the figure legends.

### Study approval.

All procedures were carried out in accordance with the *Eunice Kennedy Shriver* National Institute of Child Health and Human Development Animal Care and Use Committee guidelines and with the committee’s approval.

## Author contributions

EL designed and conducted the mouse behavioral studies; the biochemical, histologic, and molecular biology experiments; the microscopy; and the data collection and data analysis and statistics, along with preparing the manuscript. JCW designed, optimized, and performed all stereotaxic surgeries; provided guidance on data presentation and analyses; and contributed to the manuscript preparation. MB conducted the mouse behavioral studies, optimized methodologies for analyzing the *Prkar2a* localization and IEG cell counting data, and assisted with sample preparation and manuscript preparation. SB conducted the mouse behavioral studies, assisted with the related data compilation and analyses and sample preparation, and contributed to the preparation of the manuscript. CJM provided input regarding experimental design and data analyses and contributed to manuscript preparation. CAS contributed to the project and experimental design, data analyses, and manuscript preparation.

## Supplementary Material

supplemental data

## Version 1 11/03/2020

In-Press Preview

## Version 2 12/03/2020

Electronic publication
