# Supplementary material for: Loss of habenular Prkar2a reduces hedonic eating and increases exercise motivation
Source: JCI Insight. 2020 Dec 3;5(23):e141670. doi: 10.1172/jci.insight.141670 (PMC7714411; doi:10.1172/jci.insight.141670)
Supplement: supplemental data [file jciinsight-5-141670-s038.pdf]

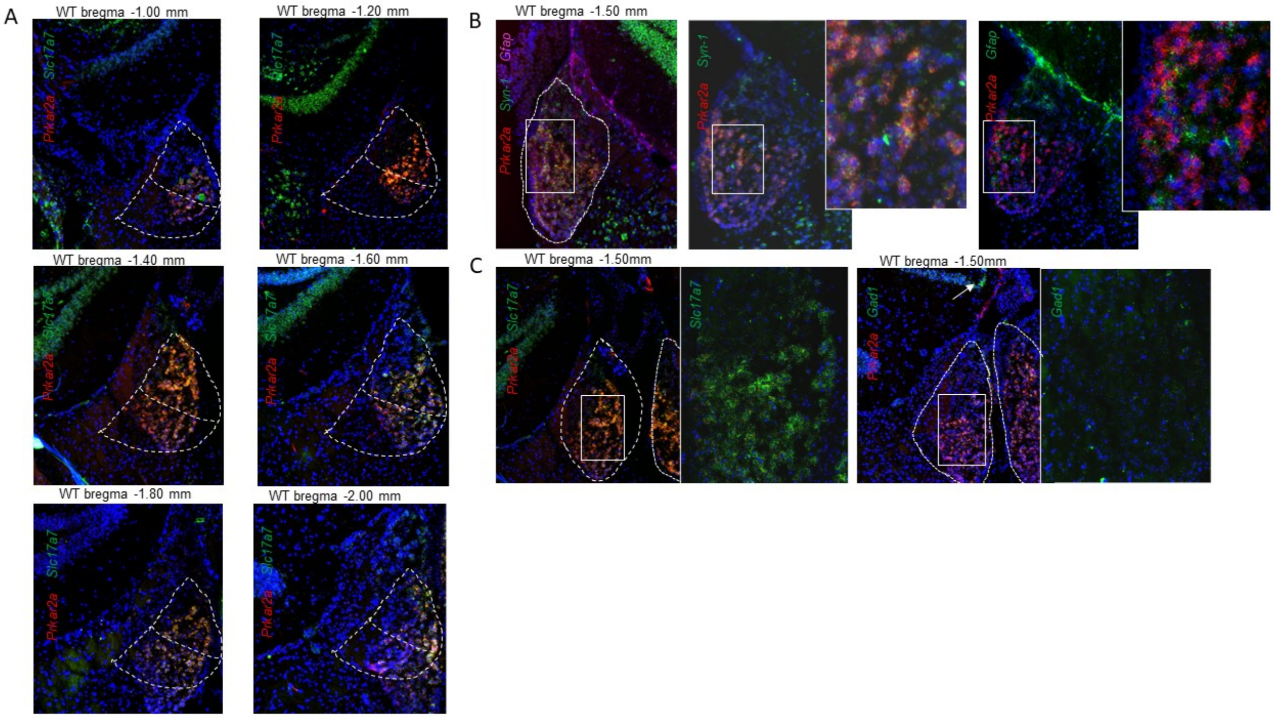

Supplemental Figure 1. Characterization of habenular *Prkar2a* expression anterior to posterior in WT C57/Bl6 mouse and general MHb cell type categorization. A. Representative *ISH* mapping images of *Prkar2a* expression in 20μM thick sections spanning the entirety of habenula defined as bregma -0.98 mm—2.06 mm.[54] Sections were also probed for *Slc17a7* as a glutamatergic marker and counterstained with DAPI. B. Representative image of MHb ISH with probes for *Prkar2a* (red), *Syn1* (green), and *Gfap* (purple) showed *Prkar2a* expression in both neurons and glial cells merged (left), *Syn1* (green) and Prkar2a (red) only (middle) and *Gfap* (green) and *Prkar2a* (red) only (right), C. ISH using probes for *Slc17a7* and *Gad1* as markers of glutamatergic or GABAergic cells, respectively was performed to confirm general cell type classification of *Prkar2a*-expressing cells in MHb.

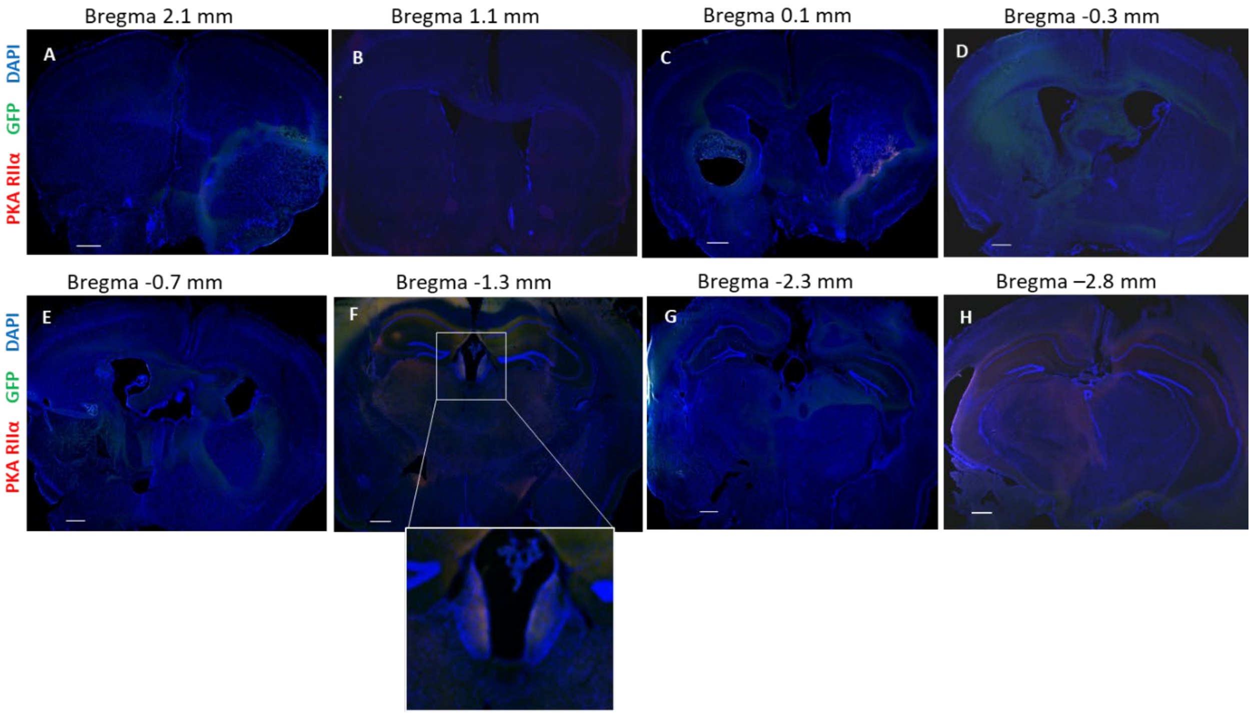

Supplemental Figure 2. Representative immunofluorescent images show that re-expression of PKA RIIα by bilateral stereotaxic injection of rAAV (pAAV-EF1a-mPrkar2a-IRES-eGFP) was successfully achieved in the MHb. A-E. Coronal sections rostral to the injection target show no expression of PKA RIIα or GFP, F. Shows re-expression of PKA RIIα (and expression of GFP), and G-H. show two show coronal sections caudal to the injection target. Scale bars represent 500 µM.

Supplemental Table 1. List of antibodies and conditions used for western blot and immunofluorescence experiments. For western blots, antibodies were diluted in 3% bovine serum albumin in 1X Tris-buffered saline with 0.1% Tween-20. For immunofluorescence of floating and mounted sections, antibodies were diluted in 3% donkey serum in 1X phosphate-buffered saline with 0.1% Triton-X 100 and 0.1% glycine.

target	primary antibody	dilution	secondary antibody	dilution
**western blot**				
DARPP-32	DARPP-32 rabbit monoclonal; Cell Signaling #2302S	1:1000	AffiniPure Donkey Anti-Rabbit IgG (H+L), HRP-conjugated; Jackson ImmunoResearch Laboratories # 711-035-152	1:10,000
DARPP-32 (T^{34})	DARPP-32 (T34) (D27A4) rabbit monoclonal; Cell Signaling #12438	1:800	AffiniPure Donkey Anti-Rabbit IgG (H+L), HRP-conjugated; Jackson ImmunoResearch Laboratories # 711-035-152	1:10,000
DARPP-32 (T^{75})	DARPP-32 (T75) (D27A4) rabbit monoclonal; Cell Signaling #2301S	1:800	AffiniPure Donkey Anti-Rabbit IgG (H+L), HRP-conjugated; Jackson ImmunoResearch Laboratories # 711-035-152	1:10,000
GAPDH	(FL-335) rabbit polyclonal; Santa Cruz # sc-25778	1:1100	AffiniPure Donkey Anti-Rabbit IgG (H+L), HRP-conjugated; Jackson ImmunoResearch Laboratories # 711-035-152	1:10,000
Histone 3	Histone 3, loading control and ChIP grade, mouse monoclonal; Abcam # ab1791	1:2000	AffiniPure Donkey Anti-Mouse IgG (H+L), HRP-conjugated; Jackson ImmunoResearch Laboratories # 711-035-150	1:10,000
HPRT	HPRT (F-1) mouse monoclonal; Santa Cruz # sc-376938	1:1200	AffiniPure Donkey Anti-Mouse IgG (H+L), HRP-conjugated; Jackson ImmunoResearch Laboratories # 711-035-150	1:10,000
PKA RIα	PKA RIα, mouse monoclonal; BD Biosciences # 610610	1:500	AffiniPure Donkey Anti-Mouse IgG (H+L), HRP-conjugated; Jackson ImmunoResearch Laboratories # 711-035-150	1:10,000
PKA Cα	PKA Cα rabbit polyclonal; Santa Cruz #sc-903	1:1000	AffiniPure Donkey Anti-Rabbit IgG (H+L), HRP-conjugated; Jackson ImmunoResearch Laboratories # 711-035-152	1:10,000
PKA Cαβγ	PKA Cαβγ (H-95) rabbit polyclonal; Santa Cruz #sc-28892	1:1000	AffiniPure Donkey Anti-Rabbit IgG (H+L), HRP-conjugated; Jackson ImmunoResearch Laboratories # 711-035-152	1:10,000

**immunofluorescence**				
c-Fos	c-Fos (4) X rabbit polyclonal; Santa Cruz # sc-52X	floating, 1:300	Donkey anti-Rabbit IgG (H+L), Alexa Fluor 555; Invitrogen # A-31572	1:400
c-Jun	c-Jun (N) X rabbit polyclonal; Santa Cruz # sc-45 X	floating, 1:300	Donkey anti-Rabbit IgG (H+L), Alexa Fluor 555; Invitrogen # A-31572	1:400
DARPP-32	DARPP-32 rabbit monoclonal; Cell Signaling #2302S	mounted, 1:500	Donkey anti-Rabbit IgG (H+L), Alexa Fluor 555; Invitrogen # A-31572	1: 500
GFP	GFP (B-2) mouse monoclonal; Santa Cruz, sc-9996	floating, 1:350; mounted, 1:400	Donkey anti-Mouse IgG (H+L) Highly Cross-Adsorbed Secondary Antibody, Alexa Fluor Plus 48; Invitrogen # A32766	floating, 1:400; mounted 1:500
MAP2	MAP-2 (AP20) mouse monoclonal; Santa Cruz # sc-32791	mounted, 1:400	Donkey anti-Rabbit IgG (H+L), Alexa Fluor 555; Invitrogen # A-31572	1:400
PKA RIIα	PKA RIIα reg (M-20) rabbit polyclonal; Santa Cruz # sc-909	floating, 1:400; mounted, 1:450	Donkey anti-Rabbit IgG (H+L), Alexa Fluor 647; Invitrogen # A32795	floating, 1:400; mounted, 1:500
PKA Cα	PKA Cα rabbit polyclonal; Santa Cruz #sc-903	floating, 1:400; mounted, 1:450	Donkey anti-Rabbit IgG (H+L), Alexa Fluor 647; Invitrogen # A32795	floating, 1:400; mounted, 1:500
PKA Cαβγ	PKA Cαβγ (H-95) rabbit polyclonal; Santa Cruz #sc-28892	floating, 1:400; mounted, 1:450	Donkey anti-Rabbit IgG (H+L), Alexa Fluor 647; Invitrogen # A32795	floating, 1:400; mounted, 1:500

[a] Blocking buffer used was 5% bovine serum albumin in 1X Tris-buffered saline with 0.1% Tween-20 (1X TBST). All other membranes, 5% nonfat milk was used in 1X TBST.

Supplemental Table 2. List of probes and dilution used for *in situ* hybridization experiments. The ACD Biotechne RNAscope Fluorescent Multiplex Assay was used after optimization for fresh frozen brain sections.

target	probe name, catalog #	dilution
***In situ* hybridization**		
Chat	RNAscope® Probe- Mm-Chat-C2, 408731-C2	1:50
Prkar2a	RNAscope® Probe- Mm-Prkar2a, 472011	1:1
	RNAscope® Probe- Mm-Prkar2a-C3, 472011-C3	1:45
Slc17a7	RNAscope® Probe- Mm-Slc17a7-C2, 416631-C2	1:50
Tac1	RNAscope® Probe- Mm-Tac1-C2, 410351-C2	1:50
Tacr1	RNAscope® Probe- Mm-Tacr1-C3, 428781-C3	1:50
Tac2	RNAscope® Probe- Mm-Tac2-C2, 446391-C2	1:50
